# IMPROVING SLEEP USING MENTORED BEHAVIORAL AND ENVIRONMENTAL RESTRUCTURING (SLUMBER)

**DOI:** 10.1093/geroni/igz038.1880

**Published:** 2019-11-08

**Authors:** Joshua Chodosh, Diana Hernandez, Michael Mitchell, Mary Cadogan, Abraham A Brody, Cathy Alessi, Jessica Smilowitz, Jennifer Martin

**Affiliations:** 1 New York University Langone Health, New York, New York, United States; 2 NYU Langone Health, New York, New York, United States; 3 VA Greater Los Angeles Healthcare System, North Hills, California, United States; 4 UCLA School of Nursing, Los Angeles, California, United States; 5 NYU Rory Meyers College of Nursing, New York, United States; 6 VA Greater Los Angeles Healthcare System, Los Angeles, California, United States

## Abstract

Sleep disturbances are common in skilled nursing facilities (SNF) affecting up to 70% of residents. Poor sleep is linked to depressed mood, cognitive impairment, increased pain, and functional disability. SNF residents depend on staff for basic day-to-day needs making it essential that staff be empowered in sleep improvement efforts. In SLUMBER, we are using a multi-site stepped-wedge design to implement a program for SNF staff to improve common sleep-disruptive factors. This three-month program includes four in-person staff meetings and three didactic webinars covering three content areas: 1) improving the nighttime sleep environment, 2) increasing daytime activities, light exposure, reducing daytime sleeping, and 3) helping individual residents having difficulty with sleep. In addition to mentoring staff on sleep improvement strategies, technology provides feedback on noise levels from decibel meters throughout the unit and weekly “sleep pearls” text messages sent to staff to reinforce teachings. We measured noise readings (in decibels) in one second increments. For sleeping hours, 10pm to 6am, we calculated the percentage of observations exceeding 60 decibels. Post intervention in the first of six study units, 78% of noise readings exceeded 60db during sleeping hours compared to three months later where 50.3% of noise readings exceeded 60db, suggesting benefits of noise-reduction efforts. SNF staff reported several instances of improving sleep among chronically poor sleepers and an improved work environment. This mentoring program can achieve important environmental improvements with perceived benefit to residents and staff. Whether this leads to objective symptom and physiological improvements awaits conclusion of this four-year trial.

